# Entropy-Based Human Activity Measure Using FMCW Radar

**DOI:** 10.3390/e27070720

**Published:** 2025-07-03

**Authors:** Hak-Hoon Lee, Hyun-Chool Shin

**Affiliations:** Department of Intelligent Semiconductors, Soongsil University, Seoul 06978, Republic of Korea; leehakhoon10@soongsil.ac.kr

**Keywords:** human activities, human motion, radar, radar signal processing

## Abstract

Existing activity measurement methods, such as gas analyzers, activity trackers, and camera-based systems, have limitations in accuracy, convenience, and privacy. To address these issues, this study proposes an improved activity estimation algorithm using a 60 GHz Frequency-Modulated Continuous-Wave (FMCW) radar. Unlike conventional methods that rely solely on distance variations, the proposed method incorporates both distance and velocity information, enhancing measurement accuracy. The algorithm quantifies activity levels using Shannon entropy to reflect the spatial–temporal variation in range signatures. The proposed method was validated through experiments comparing estimated activity levels with motion sensor-based ground truth data. The results demonstrate that the proposed approach significantly improves accuracy, achieving a lower Root Mean Square Error (RMSE) and higher correlation with ground truth values than conventional methods. This study highlights the potential of FMCW radar for non-contact, unrestricted activity monitoring and suggests future research directions using multi-channel radar systems for enhanced motion analysis.

## 1. Introduction

With the aging population and the impact of COVID-19, interest in health is continuously increasing, leading to the growth of the global healthcare market. In the future, the global trend of aging will intensify, increasing interest in personal health management [1,2,3]. Additionally, decreased physical activity contributes to a rise in the prevalence of chronic diseases such as metabolic disorders, hypertension, and cardiovascular diseases while also reducing various physiological functions. Therefore, accurately measuring and evaluating physical activity for disease prevention and health promotion are crucial for proper management [4,5,6]. Furthermore, by utilizing this information, there is potential to connect users with specialists for remote, high-quality medical services. Consequently, various types of personalized healthcare services are expected to emerge to meet these user demands [1,2].

Existing methods for measuring physical activity include gas analyzer-based (Oxycon Pro, Pennsylvania, US), activity tracker-based (ActiGraph, Florida, US), and camera-based (Microsoft Kinect, Redmond, US) techniques. Gas analyzers, which have the highest accuracy, can measure energy expenditure (EE) but are expensive and inconvenient to wear or carry. Activity trackers, which are the most common method, use triaxial accelerometers to measure activity along three axes. However, as they are attached to specific body parts such as the ankle, wrist, or waist, they only reflect activity in those areas. Measuring overall body activity requires more sensors, leading to user discomfort.

To overcome this, research on non-contact, unrestricted activity measurement has been conducted. A representative example, the camera-based method, is highly affected by lighting conditions and, most importantly, poses privacy concerns [7,8]. With technological advancements, radar sensors have become smaller, become more affordable, and significantly improved in spatial resolution. As a result, the use of radar for health monitoring has gained considerable interest, and related research is actively progressing. Radar sensors, which detect targets using electromagnetic waves reflected from them, provide a non-contact solution. Hence, radar sensors are free from the limitations of conventional sensors for measuring activity [3,7,8,9]. Research on radar sensors is being conducted in various applications, such as measuring respiration and heart rate in hospitals and vehicles, analyzing sleep patterns, detecting falls, and diagnosing movement disorders [10,11,12,13,14,15,16,17,18,19]. Initially, research on radar focused on using Continuous-Wave (CW) radar for remote heart rate and respiration measurement [10,11,12,13]. While CW radar has advantages such as low transmission power and high sensitivity, it cannot determine the distance between the radar and the subject and struggles with separating data from multipath interference and targets. To overcome these limitations, research is now utilizing Frequency-Modulated Continuous-Wave (FMCW) radar [14,15,16,17].

This study proposes an improved radar-based activity measurement algorithm using 60 GHz FMCW radar. Unlike previous research, which relied solely on distance information, the proposed algorithm incorporates both distance and velocity data to enhance accuracy. We incorporate a Shannon entropy-based metric to quantify the temporal–spatial dispersion of range changes, a velocity-weighted entropy formulation that reflects the intensity of motion, and a fully unsupervised, real-time-compatible pipeline designed for embedded radar platforms. To validate the accuracy of the algorithm, activity levels of five motion datasets are quantified and compared. The study concludes by comparing the accuracy of the proposed method with conventional methods based on motion sensor-measured activity levels (ground truth).

## 2. FMCW Radar-Based Activity Estimation Algorithm

### 2.1. FMCW Radar

**Figure 1:**
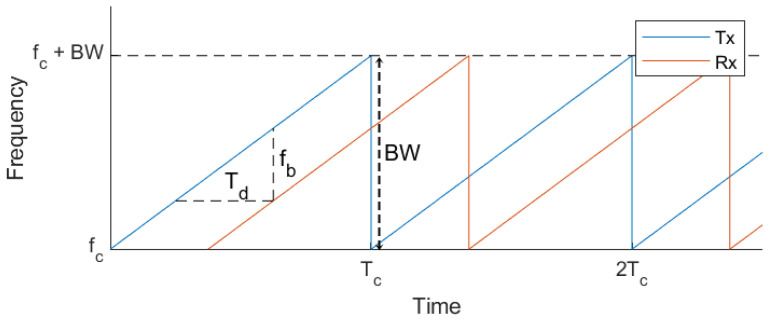
TX and RX signal of FMCW RADAR.

Radar is a sensor that transmits radio waves and receives signals reflected from objects to detect their location and velocity. The transmitted and received radar signals can vary, but this paper uses a linear-modulated FMCW radar signal as shown in Figure 1. This signal can be expressed by Equation (1). The FMCW radar may have single or multiple chirp signals for each scan. The transmission signal (TX) frequency increases linearly, and the reflected signal from the target is received as the received signal (RX). The received signal (RX) shows a delay of $T_{d}$, as can be seen in Equation (2). Here, $t_{t}$, $T_{d}$, $T_{c}$, $f_{c}$, $A_{t}$, $A_{r}$, and BW represent the transmission time, the delay time between transmission and reception, the chirp duration, the transmission frequency, the transmitted signal strength, the received signal strength, and the bandwidth of the radar signal, respectively. In a typical radar system, the distance is calculated by measuring the delay time ($T_{d}$) of the reflected signal. However, in an FMCW radar system, the distance is calculated using the beat frequency ($f_{b}$), which is the difference in frequency between the transmitted and received signals.

(1) $$s_{t}\left(t_{t}\right)=A_{t}\cdot cos\left(2\pi \left(f_{c}+\frac{BW\cdot t_{t}}{2\cdot T_{c}}\right)t_{t}\right)\;t_{t}<T_{c}$$

(2) $$s_{r}\left(t_{t}\right)=A_{r}\cdot cos\left(2\pi \left(f_{c}+\frac{BW\cdot (t_{t}-T_{d})}{2\cdot T_{c}}\right)\times \left(t_{t}-T_{d}\right)\right)\;t_{t}<T_{c},\;T_{d}<T_{c}$$

**Figure 2:**
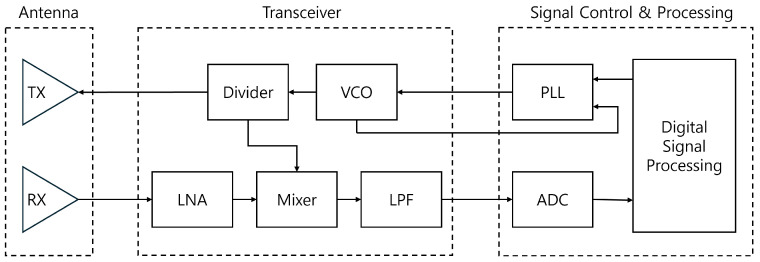
Functional block diagram of FMCW radar.

Figure 2 shows the functional block diagram of the FMCW radar. To calculate the beat frequency, the signal is demodulated through a mixer, and then only the low-frequency components, specifically the beat frequency, are extracted through a low-pass filter (LPF) and digitized via an ADC.

The demodulated radar signal is sampled at a frequency of $f_{s}$ with each chirp duration $T_{c}$ being sampled, resulting in *n* samples. Multiple chirps (*m*) are accumulated to calculate one scan (*t*). By using multiple chirps, the velocity resolution improves, and the radar becomes less susceptible to clutter. The accumulated data can be expressed by Equation (3). Here, $f_{b}$ is used to calculate the distance through Equation (4), and the time is determined by the phase difference β between each chirp signal.

(3) $$X\left(n,m,t\right)=A\left(n,m,t\right)\cdot cos\left(2\pi \cdot f_{b}\cdot n+\beta \cdot m+\phi \right)$$

(4) $$f_{b}=\frac{BW\cdot 2r}{c\cdot f_{s}}$$

(5) $$\beta =\frac{4\pi f_{c}T_{c}v}{c}$$

### 2.2. FMCW Radar Signal Processing

**Figure 3:**
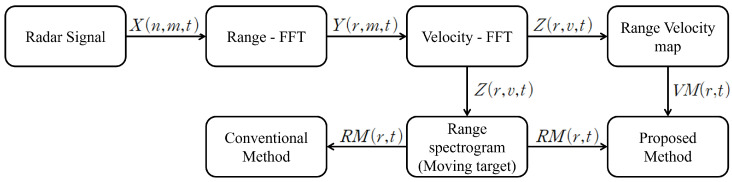
Block diagram of signal processes.

To extract distance and velocity information from the received signal Equation (3), signal processing is necessary. Figure 3 illustrates the process of handling radar-received signals. The received data undergoes a Range-FFT transformation in the n-direction to exclude symmetrical components, resulting in Equation (6), where n represents distance information. Applying Velocity-FFT in the m-direction results in Equation (7), where m represents velocity information. Accumulating the processed data into scan (*t*) creates a three-dimensional matrix containing distance, velocity, and time information.

(6) $$Y\left(r,m,t\right)=FFT_{n}\left\{X\left(n,m,t\right)\right\}$$

(7) $$Z\left(r,v,t\right)=FFT_{m}\left\{Y\left(r,m,t\right)\right\}$$

Since the resulting data is three-dimensional, it is challenging to compare changes over time at a glance. Therefore, a dimensionality reduction process is applied using Equations (8)–(10). Equation (8) sums the matrix along the velocity axis (*v*), generating a range spectrogram (RM) that captures distance changes over time. Equation (9) sums the matrix along the distance axis (*r*), forming a Doppler spectrogram (DM) that illustrates velocity changes over time. Equation (10) identifies the index with the highest magnitude along the velocity axis, displaying the highest speed at each distance in the Range Velocity Map (RVM).

(8) $$RM\left(r,t\right)=\sum_{v}Z\left(r,v,t\right)$$

(9) $$DM\left(v,t\right)=\sum_{r}Z\left(r,v,t\right)$$

(10) $$VM\left(r,t\right)=\begin{array}{c} argmax\\ v \end{array}Z\left(r,v,t\right)$$

**Figure 4:**
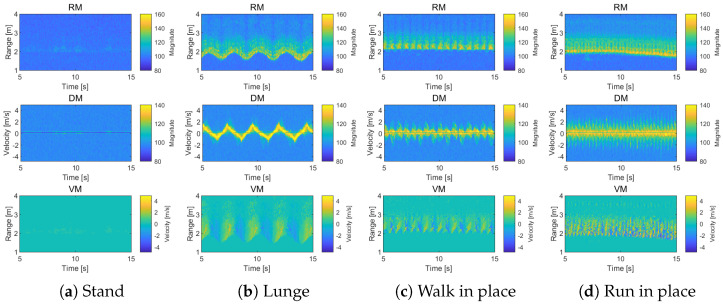
Three maps for different actions: range spectrogram, Doppler spectrogram, and range velocity map.

Figure 4 shows the results of three maps from Equations (8)–(10) for various actions such as standing, lunging, walking in place, and running in place. Figure 4a represents the standing position, where there is no movement, and in the range spectrogram, it can be observed around 2 m. Since there is no motion, the Doppler spectrogram shows almost no velocity. Figure 4b shows the lunge motion, where the entire body moves forward and backward. The range spectrogram shows back-and-forth movement around 2 m, and the Doppler spectrogram reveals positive and negative velocities alternating repeatedly. In Figure 4c, a stationary walking motion is performed. Although there is little change in distance in the range spectrogram, the Doppler spectrogram shows both positive and negative velocities simultaneously due to the foot movement. Figure 4d depicts another stationary motion, but with faster movement compared to Figure 4c. Therefore, while there is no distance change in the range spectrogram like in Figure 4c, the Doppler spectrogram shows a greater and faster change in velocity. Since the movement corresponds to both the change in distance and velocity, it is evident that both distance and velocity must be considered for activity measurement.

### 2.3. Conventional Activity Measurement Methods

The activity level estimation of the existing algorithm is calculated using the distance change obtained from the range spectrogram, as determined by Equations (8). The activity level is proportional to the degree of movement per unit time, so it is calculated using the average rate of change per time unit from the range spectrogram. To prevent the signals caused by multipath and clutter from being calculated together, even after clutter removal, a threshold $C_{th}$ is set.

**Figure 5:**
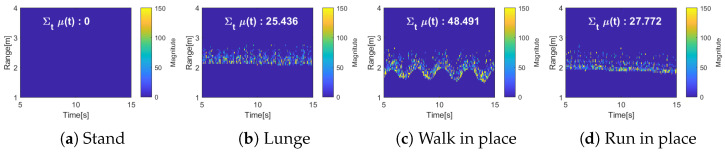
Results of the conventional method.

Figure 5 shows the results of applying the existing activity level measurement technique from Equation (11) [20].

(11) $$\mu \left(t\right)=\frac{1}{r}\sum_{r}\left\{\begin{array}{cc} {\left|RM\left(r,t\right)-RM\left(r,t-1\right)\right|}^{2} & RM\left(r,t\right)>C_{th}\\ 0 & otherwise \end{array}\right.$$

Figure 5 shows the results of applying the existing activity level measurement technique from Equation (11) to the four actions. Figure 5a represents the stationary state, so the activity level is calculated to be very low. In contrast, Figure 5b–d show relatively higher activity levels due to movement. Since only the distance change information obtained from the range spectrogram is used, the lunge movement in Figure 5b results in a higher activity level compared to the running-in-place motion in Figure 5d. Additionally, since velocity information is not considered, the activity levels for the stationary walking motion in Figure 5c and the stationary running motion in Figure 5d are calculated to be nearly the same.

### 2.4. Proposed Activity Measurement Method

**Figure 6:**
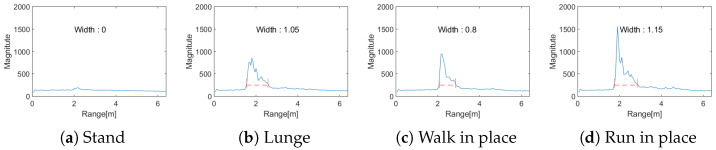
Results of the existing activity level measurement.

To overcome the limitations of the existing distance-based activity level measurement, this paper proposes a more accurate activity level measurement technique by considering not only the simple distance change but also the spatial–temporal variation in range signatures and incorporating velocity information. Figure 6 shows the distribution and range of distance changes for actions such as Stand, Lunge, Walk in place, and Run in place. The red line represents $C_{th}$ and indicates the width of the region where the values exceed this threshold. Figure 6b–d, showing the spatial–temporal variation in range signatures, is different from Figure 6a due to changes in the upper or lower body position, and the range of distance changes takes different values accordingly. As seen in Figure 6d, the range of movement change during running in place is higher than that during walking in place in Figure 6c.

In this paper, it is assumed that the degree of distance change of a motion is proportional to the activity level. The larger the distance change of the motion, the spatial–temporal variation in range signatures, as shown in Figure 6, is expected to be wider. In other words, when the activity level is high, the distribution in Figure 6 will be wider, and when the activity level is low, the distribution will be narrower. To quantify the distribution trend of almost constant changes due to movement, this paper uses Shannon entropy. Shannon entropy is a method of measuring the amount of information through the probability distribution p(i) of the information. The wider the probability distribution, the greater the uncertainty of the information, which is interpreted as a high amount of information, and it is calculated as shown in Equation (12):

(12) $$H=\sum p\left(i\right)log\left(\frac{1}{p(i)}\right)$$

To quantify the spatial–temporal variation in range signatures in the motion using Shannon entropy, a threshold $C_{th}$ is first set for the range spectrogram in Equation (8) to generate a binary spectrogram bi(r,t) as shown in Equation (13). The reason for binarizing is that the magnitude of the range spectrogram is not highly correlated with the degree of motion change. Additionally, to reflect the distribution change over time, the signal over a unit time $t_{w}$ is accumulated, and the unit time distribution is calculated as shown in Equation (14).

(13) $$bi\left(r,t\right)=\left\{\begin{array}{cc} 1 & RM\left(r,t\right)>C_{th}\\ 0 & otherwise \end{array}\right.$$

(14) $$\bar{bi}\left(r,t\right)=\sum_{t}^{t+t_{w}}bi\left(r,t\right)$$

Additionally, through the normalization process of Equation (15), a probability associated with the spatial–temporal variation in range signatures can be obtained.

(15) $$P\left(r,t\right)=\frac{\bar{bi}\left(r,t\right)}{\sum_{r}\bar{bi}\left(r,t\right)}$$

Using this, the activity level based on distance changes can be calculated as shown in Equation (16).

(16) $$H\left(t\right)=\sum_{r}P\left(r,t\right)log\left(\frac{1}{P(r,t)}\right)$$

The activity level is proportional to both the range of distance change and the velocity of the motion. Since Equation (16) does not incorporate velocity information, motions with the same range of distance change occurring at different speeds will result in the same activity level. To make the activity level proportional to velocity, we propose multiplying the magnitude of the velocity by Equation (16) and calculating the activity level as shown in Equation (17).

(17) $$\bar{H}\left(t\right)=\sum_{r}\left|VM\left(r,t\right)\right|P\left(r,t\right)log\left(\frac{1}{P(r,t)}\right)$$

**Figure 7:**
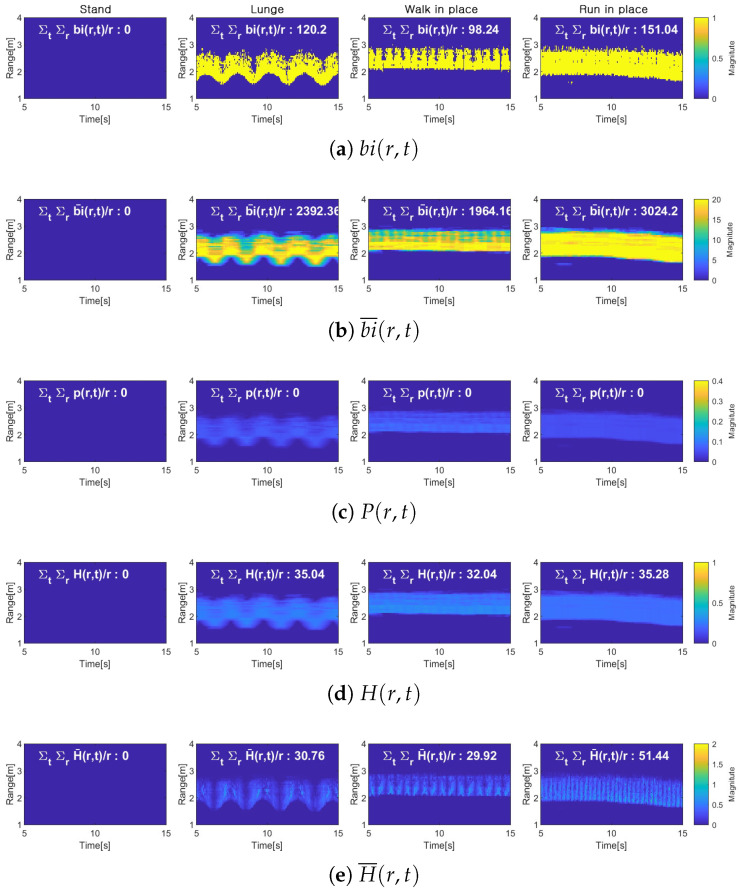
Activity measurement using proposed method for four movements.

Figure 7 shows the process of calculating activity level using the proposed method. Each subplot represents a key step in the signal processing, and the changes in the signal can be visually observed. Figure 7a shows the range spectrogram binarized into bi(r,t), illustrating the spatial–temporal variation in range signatures for the motion. Figure 7b shows the transformation into a unit time distribution by accumulating the signal over unit time tw. This process reflects the distribution of changes over unit time. Figure 7c shows the conversion process into a probability distribution P(r,t). Figure 7d presents the activity level calculation result based solely on the distance change using entropy magnitude. Finally, Figure 7e shows the result of activity level calculation by considering both distance change and velocity distance-based activity level measurement: more accurate activity level estimation can be achieved by utilizing both the spatial–temporal variation in range signatures and velocity information.

## 3. Experimental Results

The accuracy of the proposed activity measurement method is validated by using activity data obtained from motion sensors as the ground truth. Additionally, experimental results are presented to compare the proposed method with the conventional activity measurement method described in Equation (17), demonstrating the improvements achieved.

**Figure 8:**
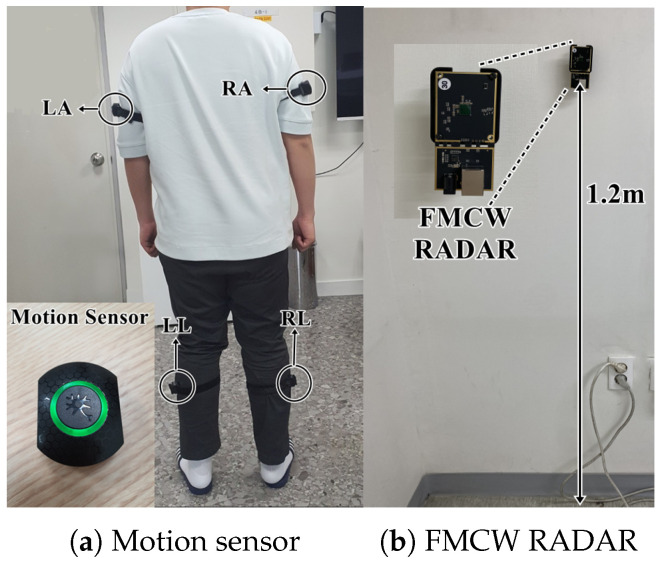
Test environment.

Figure 8 illustrates the experimental setup. The participants wear motion sensors (Model: Perception Neuron Studio, Noitom, China) on their left arm (LA), right arm (RA), left leg (LL), and right leg (RL), as shown in Figure 8a. The acceleration values along the x, y, and z axes from each sensor are combined into a single magnitude using the Signal Vector Magnitude (SVM) formula in Equation (18) [8]. The SVM values from all four sensors are then averaged using Equation (19) to obtain the overall body activity level, which is used as the ground truth.

**Figure 9:**
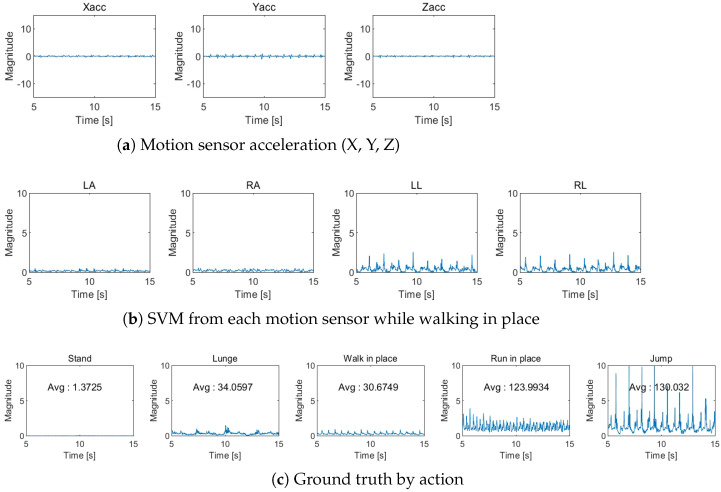
Ground truth.

Figure 9 depicts the process of obtaining the ground truth activity level using motion sensors. Figure 9a shows the calculation of SVM from the x, y, and z acceleration values using Equation (18). Figure 9b illustrates how the data from each sensor is aggregated into a single activity level using Equation (19). The ground truth activity levels for various motions are presented in Figure 9c.

(18) $$SVM\left(t\right)=\sqrt{Xacc{\left(t\right)}^{2}+Yacc{\left(t\right)}^{2}+Zacc{\left(t\right)}^{2}}$$

(19) $$SVM\left(t\right)=\frac{SVM_{la}\left(t\right)+SVM_{ra}\left(t\right)+SVM_{ll}\left(t\right)+SVM_{rl}\left(t\right)}{4}$$

The FMCW radar (Model MOD630, Bitsensing, Seongnam-si, Gyeonggi-do, Republic of Korea), measuring 9.6 cm × 6.4 cm, was mounted on the wall at a height of approximately 1.2 m and positioned to face the participant’s chest, as shown in Figure 8b. It features a field of view of ±50° in both the azimuth and elevation planes. The radar parameters are listed in Table 1, and the participants consisted of three females and five males, as shown in Table 2. Each participant independently performed five types of movements—standing, walking in place, running in place, lunging, and jumping—10 times within the radar’s detection range. The measurement duration for each movement was set to 20 s. A single-channel radar was used for the experiment.

**Figure 10:**
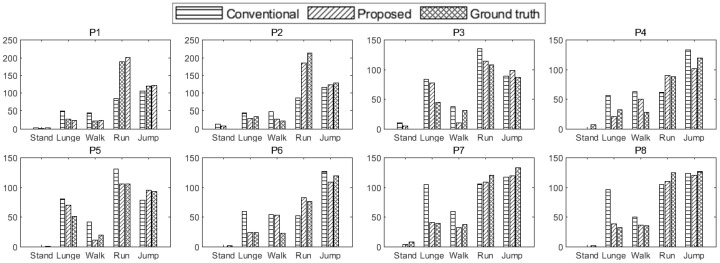
Activity measurement results for each participant.

Figure 10 compares the proposed method, the conventional method, and the ground truth activity levels, showing the average activity measurements for eight participants across different movements. The analysis results for male and female participants are presented in Table 3. For female participants, the conventional method exhibited significant errors, particularly in the running and jumping movements, with an RMSE of 55.243. In contrast, the proposed method significantly reduced the RMSE to 6.552, demonstrating improved accuracy. Similarly, for male participants, the conventional method showed high errors in walking and lunging movements, resulting in an RMSE of 23.797. However, the proposed method reduced the RMSE to 14.353, providing more accurate measurements. The overall RMSE comparison further validates the superiority of the proposed method. While the RMSE of the conventional method was 42.533, the proposed method achieved a lower RMSE of 11.157, confirming its capability for more precise activity estimation.

**Figure 11:**
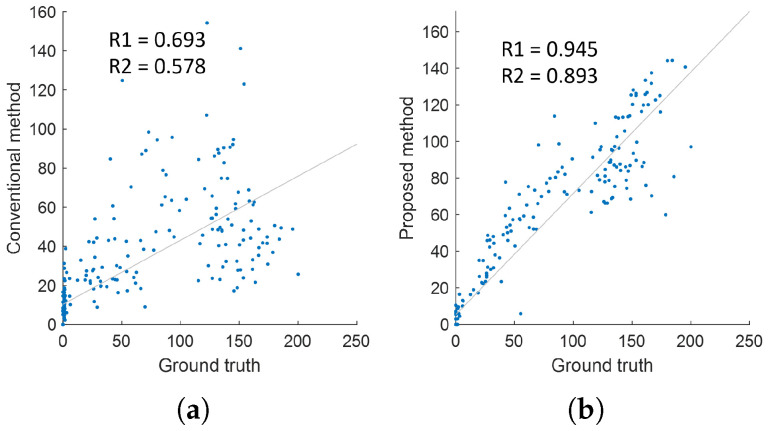
Linear regression analysis for each method. (**a**) Conventional method/ground truth. (**b**) Proposed method/ground truth.

To quantitatively analyze the performance of the proposed method, a linear regression analysis was conducted. Figure 11 presents the results of the linear regression analysis for each movement, comparing the conventional method, the proposed method, and the ground truth data. In the regression analysis, the slope of the regression line (R1) and the coefficient of determination (R2) were compared to evaluate how closely each method follows the actual measured values. Generally, a slope closer to one indicates a higher similarity to the ground truth, while a higher R2 value signifies a stronger correlation between the two variables.

Table 4 summarizes the regression analysis results by comparing the correlation coefficient (R1), coefficient of determination (R2), standard deviation, T-statistic, and *p*-value between the conventional method and the proposed method. The correlation coefficient of the conventional method was 0.693, indicating a moderate relationship with the ground truth but lacking high agreement. In contrast, the proposed method achieved a much higher correlation coefficient of 0.945, demonstrating a significantly stronger correlation and a pattern more similar to the actual data. The coefficient of determination (R2) indicates how well the regression model explains the variability in the ground truth data. The R2 value for the conventional method was 0.578, meaning it accounted for only 57.8% of the variability in the ground truth data. This suggests that while the conventional method captures certain patterns, it does not fully explain the overall data variations. On the other hand, the proposed method achieved an R2 value of 0.893, explaining approximately 89.3% of the variability, demonstrating a high level of consistency with the ground truth. Additionally, the standard deviation of the residuals, which reflects the dispersion of estimation errors, was 54.776 for the conventional method and 31.149 for the proposed method, confirming that the proposed approach yields more stable estimations. The T-statistic and *p*-value further support the statistical significance of the regression results. The conventional method showed a T-statistic of 2.688 with a *p*-value of 0.0079, indicating statistical significance at the 1% level. In comparison, the proposed method achieved a slightly higher T-statistic of 2.887 and a lower *p*-value of 0.0044, further validating the improved reliability and precision of the proposed approach over the conventional method.

## 4. Conclusions

A new method for non-contact, unrestricted activity measurement using FMCW radar is proposed. Conventional algorithms rely solely on distance information, making them unable to detect activity when there is no range variation. The newly proposed algorithm improves this by calculating entropy from distance information and incorporating velocity weighting to enhance accuracy by considering both distance and velocity data. As a result, while the conventional method maintains a certain correlation with the ground truth, it fails to fully capture variability and exhibits relatively high errors. In contrast, the proposed method shows a high correlation with the ground truth, achieving a higher correlation coefficient (*R*), determination coefficient (R2), and lower RMSE, enabling more accurate motion estimation. These results indicate that the proposed method can more precisely reflect actual activity data and has high applicability in real-world environments.

This study utilizes a single-channel radar to analyze movement based on target distance and velocity. However, since it does not provide absolute coordinate data, it has certain limitations. Although the proposed algorithm is intentionally designed as a low-complexity, real-time applicable solution based on simple heuristics, it lacks a rigorous theoretical foundation or an optimization-based framework. We need to consider an optimization framework such as machine learning-based or model-based motion scoring methods as a limitation and future direction. Also, we need low-complexity channel estimation, including considerations of Channel State Information (CSI) [21,22], which would support the generalizability of the proposed method, although the proposed method assumes stable Doppler and range estimation in typical indoor environments. To achieve more precise location estimation, a multi-channel beamforming algorithm can be applied to calculate the angle of arrival, allowing for the identification of the target’s elevation and azimuth angles. Future research is expected to leverage multi-channel radar to obtain more precise location information, refine the algorithm, and further improve activity and motion analysis accuracy. This approach will enhance the effectiveness of radar-based motion estimation techniques and expand their applicability across various domains.

## Figures and Tables

**Table 1:** Radar parameters.

MOD630 Radar		
**Parameter**	**Symbol**	**Value**
Center Frequency	$f_{c}$	60 GHz
Bandwidth	BW	3.0 GHz
Sampling Frequency	$f_{s}$	1.2 MHz
Chirp Duration	$T_{c}$	213 us
Scan Interval	$T_{s}$	50 ms
Number of chirps	*m*	64

**Table 2:** Participant physical characteristics.

Participant	P1	P2	P3	P4	P5	P6	P7	P8
Gender	F	F	F	M	M	M	M	M
Age [yr]	23	21	21	23	24	22	26	25
Height [kg]	155	158	163	167	168	171	179	182
Weight [kg]	44	48	54	75	67	72	105	100

**Table 3:** Performance comparison of activity measurement.

Gender	Method	Stand	Lunge	Walk	Run	Jump	RMSE
Female	Ground truth	1.822	24.497	23.420	202.115	121.241	-
	Conventional [20]	3.108	48.057	42.799	83.539	104.917	55.243
	Proposed	0.501	27.454	21.551	188.026	119.764	6.552
Male	Ground truth	1.273	24.240	23.224	76.076	119.613	-
	Conventional [20]	0.000	59.354	53.984	51.434	126.200	23.797
	Proposed	0.000	24.215	52.663	82.304	108.520	14.353
Overall	Ground truth	1.479	24.336	23.298	123.341	120.224	-
	Conventional [20]	1.166	55.118	49.790	63.473	118.219	42.533
	Proposed	0.188	25.430	40.996	121.950	112.737	11.157

**Table 4:** Linear regression analysis for each method.

	R1	R2	Standard Deviation	T-Statistic	*p*-Value
Conventional method [20]	0.693	0.578	54.776	2.688	0.0079
Proposed method	0.945	0.893	31.149	2.887	0.0044

## Data Availability

The data are not publicly available due to privacy or ethical restrictions.
